# PredictONCO: a web tool supporting decision-making in precision oncology by extending the bioinformatics predictions with advanced computing and machine learning

**DOI:** 10.1093/bib/bbad441

**Published:** 2023-12-08

**Authors:** Jan Stourac, Simeon Borko, Rayyan T Khan, Petra Pokorna, Adam Dobias, Joan Planas-Iglesias, Stanislav Mazurenko, Gaspar Pinto, Veronika Szotkowska, Jaroslav Sterba, Ondrej Slaby, Jiri Damborsky, David Bednar

**Affiliations:** Loschmidt Laboratories, Department of Experimental Biology, Faculty of Science, Masaryk University, Brno, Czech Republic; Loschmidt Laboratories, RECETOX, Faculty of Science, Masaryk University, Brno, Czech Republic; International Clinical Research Center, St. Anne's University Hospital Brno, Brno, Czech Republic; Loschmidt Laboratories, RECETOX, Faculty of Science, Masaryk University, Brno, Czech Republic; International Clinical Research Center, St. Anne's University Hospital Brno, Brno, Czech Republic; IT4Innovations Centre of Excellence, Faculty of Information Technology, Brno University of Technology, Brno, Czech Republic; Loschmidt Laboratories, Department of Experimental Biology, Faculty of Science, Masaryk University, Brno, Czech Republic; Central European Institute of Technology, Masaryk University, Brno, Czech Republic; Department of Biology, Faculty of Medicine, Masaryk University, Brno, Czech Republic; Loschmidt Laboratories, RECETOX, Faculty of Science, Masaryk University, Brno, Czech Republic; Loschmidt Laboratories, Department of Experimental Biology, Faculty of Science, Masaryk University, Brno, Czech Republic; Loschmidt Laboratories, RECETOX, Faculty of Science, Masaryk University, Brno, Czech Republic; International Clinical Research Center, St. Anne's University Hospital Brno, Brno, Czech Republic; Loschmidt Laboratories, RECETOX, Faculty of Science, Masaryk University, Brno, Czech Republic; International Clinical Research Center, St. Anne's University Hospital Brno, Brno, Czech Republic; Loschmidt Laboratories, RECETOX, Faculty of Science, Masaryk University, Brno, Czech Republic; International Clinical Research Center, St. Anne's University Hospital Brno, Brno, Czech Republic; Loschmidt Laboratories, RECETOX, Faculty of Science, Masaryk University, Brno, Czech Republic; Department of Paediatric Oncology, University Hospital Brno and Faculty of Medicine, Masaryk University, Brno, Czech Republic; Central European Institute of Technology, Masaryk University, Brno, Czech Republic; Department of Biology, Faculty of Medicine, Masaryk University, Brno, Czech Republic; Loschmidt Laboratories, Department of Experimental Biology, Faculty of Science, Masaryk University, Brno, Czech Republic; Loschmidt Laboratories, RECETOX, Faculty of Science, Masaryk University, Brno, Czech Republic; International Clinical Research Center, St. Anne's University Hospital Brno, Brno, Czech Republic; Loschmidt Laboratories, Department of Experimental Biology, Faculty of Science, Masaryk University, Brno, Czech Republic; Loschmidt Laboratories, RECETOX, Faculty of Science, Masaryk University, Brno, Czech Republic; International Clinical Research Center, St. Anne's University Hospital Brno, Brno, Czech Republic

**Keywords:** oncology, cancer, single-nucleotide polymorphism, personalized medicine, targeted therapy

## Abstract

PredictONCO 1.0 is a unique web server that analyzes effects of mutations on proteins frequently altered in various cancer types. The server can assess the impact of mutations on the protein sequential and structural properties and apply a virtual screening to identify potential inhibitors that could be used as a highly individualized therapeutic approach, possibly based on the drug repurposing. PredictONCO integrates predictive algorithms and state-of-the-art computational tools combined with information from established databases. The user interface was carefully designed for the target specialists in precision oncology, molecular pathology, clinical genetics and clinical sciences. The tool summarizes the effect of the mutation on protein stability and function and currently covers 44 common oncological targets. The binding affinities of Food and Drug Administration/ European Medicines Agency -approved drugs with the wild-type and mutant proteins are calculated to facilitate treatment decisions. The reliability of predictions was confirmed against 108 clinically validated mutations. The server provides a fast and compact output, ideal for the often time-sensitive decision-making process in oncology. Three use cases of missense mutations, (i) K22A in cyclin-dependent kinase 4 identified in melanoma, (ii) E1197K mutation in anaplastic lymphoma kinase 4 identified in lung carcinoma and (iii) V765A mutation in epidermal growth factor receptor in a patient with congenital mismatch repair deficiency highlight how the tool can increase levels of confidence regarding the pathogenicity of the variants and identify the most effective inhibitors. The server is available at https://loschmidt.chemi.muni.cz/predictonco.

## INTRODUCTION

In 2020 alone, more than 19 million cases of cancer were diagnosed, of which 10 million were mortal [1, 2]. Cancer has a projected load of 28.4 million cases in 2040 [2]. Cancer treatment is generally based on the following three traditional approaches: operative removal via resection/excision surgeries, radiotherapy and chemotherapy. These traditional treatment options tend to have higher mortality rates as compared to personalized (precision) medicine-based techniques, which match the right drugs to the right patients [3].

Personalized medicine-based techniques have their limitations. Decisions are often based on next-generation sequencing technologies, which can generate a large amount of genomic or transcriptomic data. It can be difficult to analyze and interpret this amount of data in a clinically feasible manner. This leads to a divide between the generation of experimental data and their application during the decision-making process. For example, when targeted next-generation sequencing panels are used for analyzing multiple genes simultaneously, various coding sequence mutations are typically found. And when no previous data on the effect of such mutations on the expressed protein are available, the data cannot effectively support decisions since there is usually little space to study each mutation in sufficient detail [4].

With the recent advances in protein modelling [5, 6], e.g., in prediction of the effect of single missense mutation on a protein structure [7], stability [8, 9], function [7] and protein–ligand interactions [10], much more information can be harvested from exome sequences of mutated proteins. Computed and carefully interpreted information can be used for making high-quality, well-informed decisions. Furthermore, the data retrieved from protein structures may be coupled with additional analyses, such as the virtual screening of drug libraries [11]. Comparative analysis may be carried out if the calculations are performed in the same conditions on both the wild type(s) and the mutated protein(s).

On the one hand, the traditional method of deliberation and discussion by multiple experts is naturally time-consuming. On the other hand, an alternative consisting of performing all the aforementioned analyses requires significant knowledge of computational biology and bioinformatics. It would also entail considerable time if performed manually. Here, we introduce PredictONCO, a web server for fully automated and fast analysis of the effect of mutations on protein structure, stability and function in 44 known protein targets relevant for oncology. PredictONCO applies computational methods and bioinformatic analyses and produces actionable reports within a few days. To ensure the high quality of the results, the entire pipeline was thoroughly validated using 108 clinically and experimentally characterized mutations covering all 44 proteins. The final report includes visualizations to help with data comprehension.

## WORKFLOW

**Figure 1:**
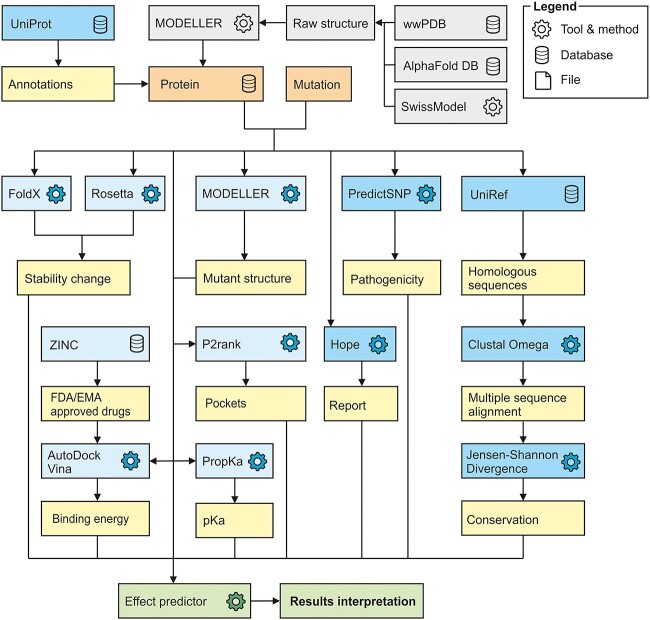
The workflow of PredictONCO. The only required input is a protein and a mutation (orange boxes). The grey boxes show pre-treatment steps that were done manually in advance to prepare high-quality protein structures as a reliable starting point for the calculation. Once the calculation is submitted, multiple analyses are executed. The sequence-based analyses (dark blue boxes), such as annotations gathering, pathogenicity prediction, conservation prediction and HOPE, are performed for all of the mutations. For mutations in the catalytic domain with available 3D structure, also structure-based analyses (light blue boxes), such as stability and pKa prediction, pockets detection and virtual screening, are performed. Once all the results are available, the effect of the mutation is predicted using our in-house machine learning predictor, and the results can be visualized and explored (green boxes). The yellow boxes briefly describe outputs collected from each analysis. The entire pipeline and all the individual steps are described in more detail in the Workflow section.

The workflow of PredictONCO consists of two major steps: (i) selection of target protein and mutation and (ii) data analysis (Figure 1). In the first step, the users are requested to select a protein of their interest from the list of 44 cancer-associated drivers and targets (Supplementary Material) and specify a mutation for its analysis. In the second step, the calculations are executed consecutively, and users are provided with results for an interactive and visual inspection and analysis.

### Mutation selection

The first step of the workflow is the specification of an input as one of the 44 pre-defined oncology-relevant proteins (Supplementary Material). Clinicians carefully selected these proteins with the aim of covering the majority of common oncogenic drivers and therapeutic targets. The upload of custom structures is currently not supported in our pipeline. The rationale for this decision is that such input would incur a severe risk of biological misinterpretation of the obtained results. Most oncology-relevant targets are complex transmembrane proteins, for which a single and reliable experimental structure that can be used for analyses without extensive manual processing is generally unavailable. Therefore, adding a new target requires non-trivial manual curation and integration of knowledge from multiple bioinformatics strategies. To increase the applicability of PredictONCO, we offer the opportunity to request the addition of a new oncology-related protein to the list of targets. The advantage of this approach is that all targets will be carefully prepared and thoroughly validated.

All of the 44 proteins available to the users were previously curated, following a number of steps. The most suitable calculation parameters were optimized for each target to increase the reliability of the results. First, the protein sequence and annotations were fetched from the UniProt database [12]. The essential residues were then re-confirmed in literature. For cytoplasmic units, the best available experimentally derived structure was selected from the wwPDB database [13] and the residue indices were mapped using the SIFTS database [14]. In the case of two proteins, there was no experimental structure available. For the first one (PDGFRβ), the structure was fetched from the AlphaFold database [15]; for the second one (VEGFR3), the structure was modelled using the SWISS-MODEL web server [16]. After that, all structures were semi-automatically processed to treat unnatural amino acids, remove non-co-factor ligands and improve their quality by modelling missing parts of the structure and reverting mutations using MODELLER [5]. As the last step, homologous sequences with sufficient identity (more than 50%) and coverage (±20% of the query sequence length) were downloaded from the UniRef database [17], and a multiple sequence alignment was generated using Clustal Omega [18] from EMBL-EBI web services [19]. This alignment was further used for conservation analysis using the Jensen–Shannon divergence algorithm [20] and transformed to mutability grades by using HotSpot Wizard [21] thresholding. All these steps are pre-calculated for each of the 44 listed curated proteins.

Once the protein is selected, users are requested to specify the mutation by typing in the substitution or selecting the position of the substituting amino acid using a visualized protein sequence. These calculations usually take up to 48 h, depending on the protein sequence length and other structural parameters (could be longer under heavy workload of the server). This time threshold was carefully selected as a balanced spot between time constraints of medical boards and the possibility to use the most reliable and accurate computational methods. However, all finished calculations are stored in a database and when a previously computed mutation is requested, the calculation output is immediately served from saved results. All 913 mutations used during the training and testing of the predictor (see The Mutation Effect Predictor) are pre-calculated in such fashion. Optionally, the users can also provide the job title and email address, which will be used to deliver alerting emails about initiating and completing the calculations, links to relevant output pages and the reference to be cited whilst publishing the results obtained using the PredictONCO server.

### Data analysis

Once the job is submitted, all calculations are executed automatically. The specific analyses depend on the region where the mutation is located and on the availability of a structure information for that region. If a high-quality 3D structure is available and the mutation is located in the cytoplasmic domain responsible for ligand binding, all analyses are performed, including the function-related and drug-related calculations to assess the potential inhibiting role of small molecules. The function-related and drug-related calculations are omitted if the mutation is in the extracellular unit. Only sequence-based analyses can be performed for the transmembrane and undefined regions, as these regions often lack the experimental structural information required for precise force-field calculations. Finally, the effect of the mutation is predicted using a newly developed machine learning-based predictor (see The Mutation Effect Predictor).

#### Structure analyses

Initially, a mutant structure is constructed using the MODELLER [5] software. The mutant structure is used to check any possible disruptive changes in the structure and to perform a comparative analysis against the wild-type structure. Then, the effect of a mutation on protein stability is estimated using Rosetta ddg_monomer [9] and FoldX [22]. Function and drug-related properties are predicted in the case the mutation is located in the cytoplasmic domain. These include calculating the p*K*_a_ change of catalytic residues using PROPKA3 [23] and identifying residues forming the ligand-binding pocket using P2Rank [24]. In the last step, only in the cases required (the mutation and ligand binding site located in the cytoplasmatic domain), the virtual screening of a large dataset (4380 small molecules) of Food and Drug Administration (FDA)- and European Medicines Agency (EMA)-approved drugs extracted from the ZINC database [25] is performed using AutoDock Vina [11].

#### Sequence analyses

The most important sequence analysis is a prediction of an effect of a mutation on a protein function performed by the meta-server PredictSNP [7], which combines six established tools (MAPP [26], PhD-SNP [27], PolyPhen-1 [28], PolyPhen-2 [29], SIFT [30] and SNAP [31]) into a single prediction. Other important values, such as an estimation of potential local changes induced by the placement of different amino acids, are calculated using the HOPE server [32].

#### Database mining

The next step is the extraction of important and relevant information from publicly available database. We use the UniProt database [12] as the primary source, which provides an exhaustive amount of data aggregated from multiple sources. Data fetched from UniProt are heavily filtered out to retain only information related to diseases, known clinically relevant drugs and known mutations.

#### The mutation effect predictor

In the end, all obtained values from the structure and sequence analyses were used as features for our newly trained predictor: essentiality of the mutated residue (yes—1/no—0), the conservation of the position (the conservation grade and score), the domain where the mutation is located (‘cytoplasmic’, ‘extracellular’, ‘transmembrane’, ‘other’—one-hot encoded), the PredictSNP score, the number of essential residues, FoldX and Rosetta ddg_monomer scores, whether the residue is in the catalytic pocket (yes—1/no—0) and the pKa changes (the minimum, maximum changes and the number of essential residues whose pKa was changed). The predictor is based on the XGBoost classification model and returns the probability of the oncogenic effect of a mutation. This model consists of a collection of small decision trees, trained sequentially by fitting each one of them to the gradients of the loss function from the previous iteration. On every iteration, the predictions of the current set of decision trees (the probabilities of a mutation being oncogenic) are weighted and evaluated against the ground truth to calculate the loss function. Therefore, even despite having weak predictive power separately (each decision tree typically uses one to three features only), when combined they often show state-of-the-art performance, comparable with advanced methods from deep learning.

**Figure 2:**
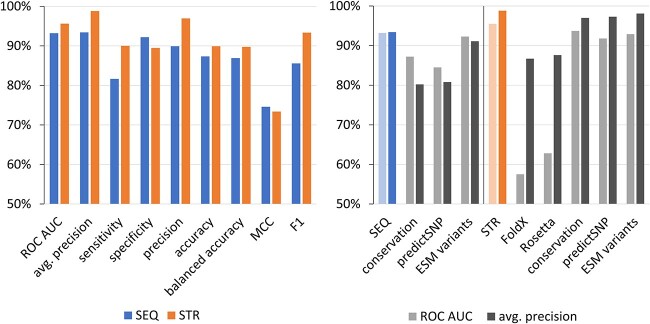
The performance of the structure-(STR) and sequence-based (SEQ) predictors on the held-out test set of 213 and 89 mutations, respectively. Left: The area under the receiver operating characteristic curve (ROC AUC) and average precision values show strong performance for the probability of the oncogenic effect of a mutation returned by the predictors. The remaining values were calculated for the cut-offs of 0.50 applied to this probability, corresponding to the maximum of the Fowlkes–Mallows index. Right: The comparison to the individual tools and the state-of-the-art method ESM variants according to ROC AUC and average precision metrics show overall better performance in both SEQ and STR evaluations.

The predictor was developed using a dataset of 509 oncogenic and 564 non-oncogenic mutations, 377 and 76 of which, respectively, had structural information as described previously. All mutations were compiled from the ClinVar [33] and OncoKB [34] databases and annotated with a clinically verified effect based on the available information in these and other precision oncology databases [35–38] and primary literature. The resulting predictor was validated on the independent subset of 20% held-out data, grouped by mutated positions to ensure that positions in the test set do not appear in the training set (213 mutations for the sequence-based and 89 mutations for the structure-based predictions; for more information on the dataset, see https://zenodo.org/records/10013764), showing the areas under the receiver operating characteristic curve (ROC AUC) of 0.96 and 0.93 and the average precision from the precision–recall curve of 0.99 and 0.93 for the structure-based and sequence-based predictions, respectively (Figure 2). The performance of the predictor on the corresponding test set was also compared with individual tools (PredictSNP, conservation score, FoldX, Rosetta) as well as the recently published state-of-the-art tool ESM variants for predicting the disease variant effects [39]. More details about the predictor training and testing are available at https://loschmidt.chemi.muni.cz/predictonco/help.

## DESCRIPTION OF THE WEB SERVER

### Input

**Figure 3:**
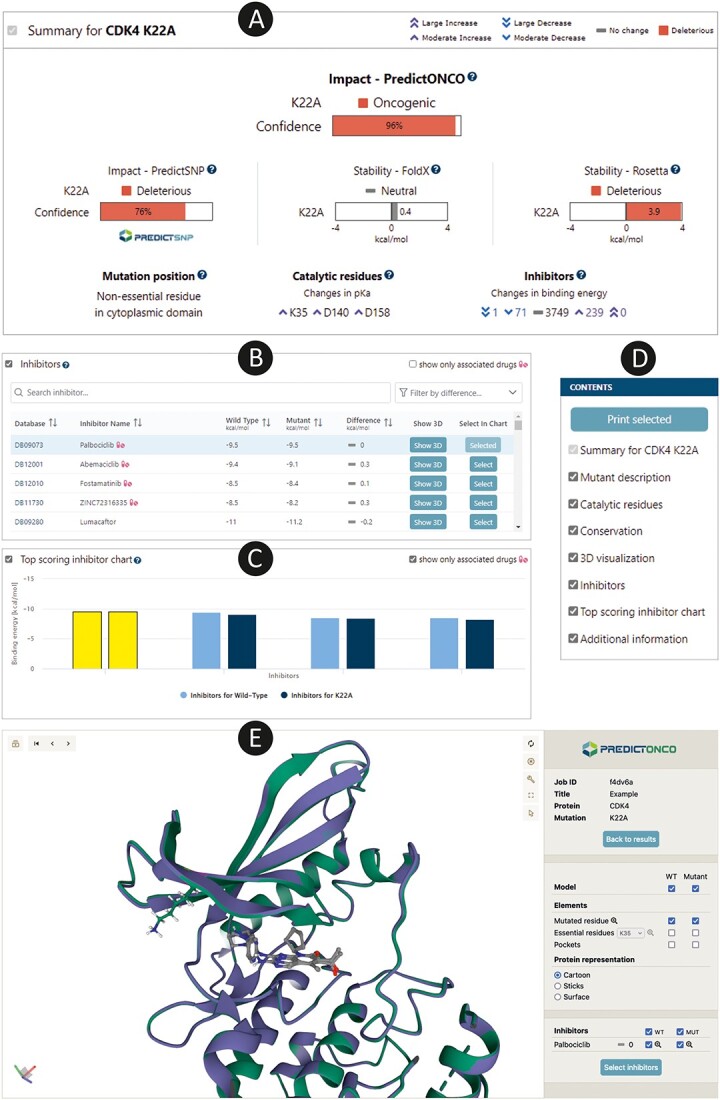
The PredictONCO graphical user interface displays results for the case study on CDK4 mutation K22A. (**A**) The Summary section provides the final impact prediction accompanied with a visual representation of the most important data collected for a given mutation. (**B**) The Inhibitors table displays a comprehensive list of binding energies for 4380 FDA/EMA-approved inhibitors with a special label for those with known association with the given protein. (**C**) Inhibitors chart is a visual representation of binding energies for the top hits. (**D**) The Contents panel enables quick navigation through the results page. (**E**) Structure viewers provide interactive 3D insight into the mutant and wild-type structures, their differences and binding poses of inhibitors.

PredictONCO requires only two inputs from the users—a protein and a mutation (Figure 3). The protein selection is made from a comprehensive table of 44 proteins (Supplementary Material). Each protein is represented by its most important identifiers, such as the gene name, protein code, UniProt accession id and protein name. The table is sortable by any of its columns and can be interactively searched. Furthermore, details about each protein can be displayed with a single click on the ‘>’ button on the left. These include detailed information about the catalytic function and regulation and preview images of the available structures for all the units. The second required input is a mutation. It can be selected either by quick textual input or interactive selection of a position and target amino acids from a protein sequence preview. In both cases, the user gets immediate feedback if there is any problem with the input, such as when a wrong position or amino acid is selected. Optionally, users can provide their email addresses to be notified about the job status and give the job a custom title. The last step is the confirmation of the user’s academic status required by licenses of some of the integrated tools (MODELLER, Rosetta and FoldX). Some analyses will be excluded for commercial users if the company does not own a proper license.

### Output

After the job submission, the user is redirected to the results page (Figure 3). If the same mutation was already analyzed in a previous job, the data are retrieved from the library of finished jobs, and all results are shown immediately. Otherwise, the calculations are started, and the results appear continuously whenever individual calculations are finished. To improve the users' experience, a panel with a list of sections (Figure 3D) on the left allows quick navigation amongst the analyses. Moreover, the sections can be interactively collapsed to allow the user to create a customized, cleaner view of the results. As the tool is designed mainly for users without advanced bioinformatics knowledge, most details and numerical values are hidden and represented by a unified system of five categorical labels—Large Increase, Moderate Increase, No change, Moderate Decrease and Large Decrease. In the cases where further explanation or context is needed, the results are provided in the form of easily understandable sentences. This makes data interpretation possible even without understanding the underlying values, which was requested particularly by the users from the medical community. However, all the computed values can still be displayed by checking show details. Moreover, in the results section, the emphasis lies on the analysis of the differences between the mutant and the wild-type protein. This helps to focus the users’ attention on what is more scientifically relevant: the effects caused by the mutation rather than absolute values. Still, the values for both the wild-type and the mutant protein can easily be displayed.

The results are grouped into eight main sections. The most relevant information is displayed in the Summary (Figure 3A), which provides a quick and comprehensive overview of the data. The key information shown is the overall predicted effect of the mutation and the easy-to-understand description of the mutation location and residue importance. Furthermore, it displays the probability of damaging the protein function, conservation score, estimation of changes in the protein stability, changes in p*K*_a_ of catalytic residues and an overview of changes in the binding energies of drugs. Also important amongst results is the Inhibitors section (Figure 3B), which displays the binding energies of all potential drugs from the FDA/EMA-approved database in a tabular format. This table is also sortable and fully searchable, which assists in the identification of the most suitable drugs for repurposing. Furthermore, all drugs with a known association with the target protein are labelled using a special icon to indicate their importance. For an easier and more comprehensive comparison, binding energies can also be explored in the form of an interactive chart below the table (Top scoring inhibitor chart, Figure 3C). The remaining sections provide details about various important properties, such as a description of differences between the properties of wild-type and mutant amino acids (Mutant description, Catalytic residues); assessment of conservation of mutated position (Conservation); interactive 3D visualization of the protein structure with highlighted mutation using the Mol* Viewer [40] (Structure viewer, Figure 3E); and information extracted from the UniProt database about function, interactions and known involvement in diseases (Additional information). To save the results for later use, users can either print the currently displayed view of the results or download them as a PDF report. Moreover, a detailed table of inhibitors is also available for download.

### Server testing and performance

The robustness of the calculations was tested by analyzing a set of 1073 mutations used for the development of the mutation effect predictor. Moreover, 108 mutations covering all target proteins were used for thorough validation of obtained results. Seventy-seven of 108 jobs were run on parts of protein sequence for which structural information was available. All 1181 jobs finished without errors. The lengths of the calculation varied between 1 and 10 hours.

## USE CASE 1: ANALYSIS OF CYCLIN-DEPENDENT KINASE 4 K22A MUTATION IN A PATIENT WITH MELANOMA

Cyclin-dependent kinase 4 (CDK4) is a crucial molecule mediating the cell cycle [41]. The *CDK4* gene is frequently altered in a wide spectrum of malignancies, including lung adenocarcinoma, tumours of the central nervous system or melanoma [42]. The most commonly observed alteration is CDK4 amplification. However, several point mutations resulting in non-synonymous amino acid changes have also been reported and can present a challenge for interpretation and treatment [43–45].

The tumour-derived DNA from a patient with melanoma was analyzed by whole-exome sequencing, and amongst other findings, CDK4 K22A alteration was identified. The mutation K22A previously showed to significantly decrease CDK4 binding to cyclin D1 and tumour suppressor p16 [46, 47] but no clinical evidence of mutation effect is available. Given the known significance of CDK4 in melanoma, evaluating its deleteriousness from sequential and structural perspectives is required to assess its clinical potential further. The effect of the mutation on protein function was assessed as deleterious with a confidence score 76% if only bioinformatic predictive algorithms within PredictSNP were applied. Once evaluated comprehensively by PredictONCO with the inclusion of advanced computational tools, the confidence level increased to almost 100%; therefore, the mutation impact points towards a significant pathological effect (Figure 3). Stability prediction by Rosetta shows a destabilizing effect of the mutation K22A on protein structure (energy change +3.2 kcal/mol), also suggesting its importance in tumour pathophysiology. This residue is not essential for function, and changes in ionization constants p*K*_a_ of catalytic residues are predicted to be negligible. Based on visualization, we can observe that the mutation is on the edge of the binding site and can have some effect on the interactions with critical molecules. Moreover, the protein loses an important interdomain salt bridge interaction. Therefore, an impact on the structure and/or substrate binding affinity can be expected based on these analyses.

For therapeutic implications, it is essential to have an overview of existing inhibitors that can target the studied protein and see if the mutation causes any differences in their binding energy estimated by molecular docking calculation. Proteins can be generally targeted by multiple inhibitors. However, only a few of them represent FDA/EMA-approved therapeutics for oncology use. PredictONCO identified four FDA/EMA-approved inhibitors of CDK4, with ribociclib (labelled as ZINC72316335 in the table) and palbociclib being the most commonly used. In the case of K22A, all four associated drugs show no significant difference between affinity in wild-type and mutant proteins. According to the docking results, none of the four drugs is directly influenced by the mutation, and their inhibitory effect should not be compromised. The highest affinity of the four associated drugs was observed for palbociclib (*E*_bind_ = −9.5 kcal/mol), and, therefore, it is the top hit for potential treatment. Moreover, several other drugs were predicted as even better binders, with lumacaftor (*E*_bind_ = −11.2 kcal/mol) being the top hit indicating a potential for the drug repurposing strategy (Figure 3).

In summary, the results contributed to evaluating the effect of the mutation. Several parallel lines of evidence obtained from PredictONCO calculations and database searches suggested that the mutation K22A significantly affects protein structure and function and can be related to tumour pathophysiology with high probability. The PDF report with all results for the K22A mutation is available in the Supplementary Materials.

## USE CASE 2: ANALYSIS OF ANAPLASTIC LYMPHOMA KINASE 4 E1197K MUTATION IN A PATIENT WITH LUNG CARCINOMA

In a patient with EML4::ALK rearranged non-small cell lung carcinoma initially treated with crizotinib, sequencing of tumour tissue resected upon tumour progression was performed to search for a potential cause of treatment failure. The data analysis revealed the presence of the anaplastic lymphoma kinase 4 (ALK) E1197K substitution. Since there is only limited evidence regarding the variant’s significance in the clinical databases of genetic variants, PredictONCO analysis was performed to evaluate the variant’s impact. The prediction results suggested that the variant is oncogenic with a 100% confidence score, and it affects the protein stability as predicted by both FoldX and Rosetta. Within the provided summary, substantial differences between the wild-type and mutant residue in their size and charge and a high level of evolutionary conservation of the wild-type residue are highlighted, underscoring the potential implications of this substitution. In terms of therapeutic options, the binding energies of second- or third-generation inhibitors such as ceritinib, alectinib and lorlatinib, which could be considered for subsequent treatment, do not appear to be significantly affected by the presence of the variant. This suggests that these inhibitors may remain viable options for following clinical management.

The PDF report with all results for the E1197K mutation is available in the Supplementary Materials.

## USE CASE 3: ANALYSIS OF EPIDERMAL GROWTH FACTOR RECEPTOR V765A MUTATION IN A PATIENT WITH CONGENITAL MISMATCH REPAIR DEFICIENCY

The DNA from a tumour of patient with congenital mismatch repair deficiency (CMMRD) was subjected to NGS analysis. CMMRD, characterized by a disruption in one of the fundamental DNA repair mechanisms, led to the presence of hundreds of variants in the tumour DNA. These variants often possess unknown functional significance, as they are randomly scattered throughout the DNA sequence [48]. Patients with CMMRD-driven tumours are often successfully managed using cancer immunotherapy [49]. However, if patients experience disease progression or recurrence, somatic NGS data might be utilized to evaluate whether there are additional signs of specific signalling pathway activation that could be therapeutically explored. In this specific patient, the epidermal growth factor receptor (EGFR) V765A variant was found together with many other tumour-associated alterations, representing a potential predictive biomarker for RTK (receptor tyrosine kinases) inhibitor administration despite very limited clinical data on its functional impact. To determine whether further investigation was warranted and to comprehensively evaluate available *in silico* evidence, PredictONCO analysis was conducted. The overall assessment returned a benign prediction with a 55% confidence score. In alignment with the overall prediction, PredictSNP and FoldX deemed this mutation to have a neutral impact on both sequential and structural levels, respectively. Stability prediction performed by Rosetta yielded discordant results, indicating a slightly deleterious effect on protein stability. In terms of evolutionary conservation, the residue displayed no significant conservation across species. When considering the entirety of the PredictONCO analysis, it did not corroborate the potential impact of the EGFR V765A variant. Consequently, this variant was not pursued further as a predictive biomarker.

The PDF report with all results for the V765A mutation is available in the Supplementary Materials.

## CONCLUSIONS AND OUTLOOK

PredictONCO 1.0 is a novel web server for rapid analysis of the effects of missense mutations detected by exome sequencing in tumour tissue. It enables the analysis of important protein targets with a known relation to oncological diseases. The tool covers several important areas of analysis: (i) computational analysis of sequential and structural properties of the mutant enzyme, (ii) virtual screening of 4380 FDA/EMA-approved drugs and (iii) verified knowledge aggregated in the UniProt database. To make the tool accessible to researchers outside the bioinformatics community, the graphical user interface was designed to be highly interactive and visualize the critical information obtained from computations. PredictONCO provides the data in an easy-to-understand format, using various visual aids and comprehensible language. To meet the demands for a short execution time required by clinical practice, only the tools with high predictive accuracy and low computational time were selected, and they are executed in a highly parallel manner using our high-performance computing environment. Moreover, all previously calculated results are stored for their immediate re-use. To ensure the quality of provided data and prediction, the server was thoroughly validated using 108 experimentally characterized mutations with known clinical association with tumour.

One of the limitations of the current version is its limited ability to target the mutations causing loss of function. Even though these mutations can be successfully identified during the calculations, the server itself cannot provide actionable insight into the treatment strategy. Repurposing the drugs, which are mostly inhibitors, on an already non-functional protein will not bring helpful effects. To overcome this limitation in the next version, we plan to incorporate knowledge from the biological pathways that will also allow inhibiting proteins downstream in the pathway. The second drawback of our method is its inability to predict effect of insertions and deletions even though they are also often related to oncological diseases. Unfortunately, this problem is still generally unresolved and there are no reliable tools available. However, we are actively monitoring newly published tools, and as soon as any promising options emerge, we will work to integrate them into PredictONCO. The third challenge concerns missing and low-quality regions of protein structures. We have already attempted to use models that AlphaFold 2 [15] predicted, but faced problems with incorrectly modelled transmembrane regions. Continued advancement in structural biology can potentially address this issue in the future. The last improvement, closely related to the lack of structures, is the support of processing user-uploaded structures. We want to implement a highly robust solution to secure that an uploaded and curated protein structure is correct for calculating biologically meaningful results.

**Key Points**

- PredictONCO is a web server that analyzes the effects of mutations on proteins frequently altered in various cancer types.
- The server assess the impact of mutations on the protein sequential and structural properties and apply a virtual screening to identify potential inhibitors that could be used as a highly individualized therapeutic approach.
- PredictONCO integrates predictive algorithms and state-of-the-art computational tools combined with information from established databases.
- The server currently covers 44 common oncological targets and provides a fast and compact output ideal for the often time-sensitive decision-making process in oncology.
- Three use cases of missense mutations highlight how the tool can increase levels of confidence regarding the pathogenicity of the variants.

## Supplementary Material

report_ALK_E1197K_bbad441

list_of_protein_targets_bbad441

## Data Availability

PredictONCO is a web server available at https://loschmidt.chemi.muni.cz/predictonco/. Training and test datasets are available at https://zenodo.org/records/10013764.

## Author Biographies

**Jan Stourac** is a doctoral student and software engineer in Loschmidt Laboratories at Faculty of Science at Masaryk University and International Centre for Clinical Research. He is focused on the development of user-friendly web servers and databases for protein engineering such as HotSpot Wizard, PredictSNP, Caver Web and FireProt.

**Simeon Borko** is a software engineer within the bioinformatics team of Loschmidt's laboratories at the Faculty of Science at Masaryk University, where he is involved in the development of several bioinformatics tools including FireProt, PredictONCO, and Caver web.

**Rayyan T Khan** is a doctoral student in Loschmidt Laboratories at Faculty of Science at Masaryk University. His research focus is ancestral sequence reconstruction and computational personalised oncology. He was also involved in a development of FireProt-ASR web server.

**Petra Pokorna** is a molecular biologist within the Ondřej Slabý research group at the Central European Institute of Technology at Masaryk University in Brno, Czech Republic. Her main research focus is on high-throughput genomic technologies and their application to the diagnostics of solid tumors and rarediseases.

**Adam Dobias** was a master student of Bioinformatics programme at Faculty of Science as Masaryk University. He is interested in development of bioinformatics tools and services.

**Joan Planas-Iglesias** is a post-doctoral researcher at the Loschmidt Laboratories affiliated with the Department of Experimental Biology of Masaryk University and the International Clinical Research centre of St. Anne's University Hospital in Brno, Czech Republic. His research is focused in the study of protein flexibility and fragment-based design of proteins.

**Stanislav Mazurenko**, PhD, is the leader of the Artificial Intelligence in Protein Engineering team of Loschmidt Laboratories, whose goal is toleverage available biological data to create reliable and interpretable tools for the analysis and design of improved protein variants.

**Gaspar Pinto** was a postdoctoral fellow in Loschmidt Laboratories at Faculty of Science at Masaryk University. His research focus is mostly molecular dynamics and quantum mechanics simulations for protein engineering and drug design.

**Veronika Szotkowska** is anundergraduate student of Mathematical Biology programme at Faculty of Science, Masaryk University. She is interested in molecular modeling and in silico analysis of proteins.

**Jaroslav Sterba** is a Professor of Oncology and the head of the Department of Pediatric Oncology at the University Hospital in Brno, Czech Republic. He is also a chair of the Czech Pediatric Oncology Society and a member of the Paediatric Committee of the European Medicine Agency. His scientific interests include metronomic treatment and targeted treatment within the precision oncology paradigm of high-risk pediatric solid tumor patients.

**Ondrej Slaby** is a Professor of Medical Chemistry and Biochemistry and a group leader at the Central European Institute of Technology and the head of the Department of Biology for the Faculty of Medicine at Masaryk University, and the head of the Laboratory of Molecular Pathology at the Department of Pathology, University Hospital Brno. His research group is focused on comprehensive genomic profiling for individual therapeutic planning in high-risk pediatric solid tumors, as well as the roles of non-coding RNAs in the molecular pathology of solid cancers and their potential as innovative diagnostics and novel therapeutic targets in oncology.

**Jiri Damborsky** is the Josef Loschmidt Chair Professor of Chemistry and Professor of Biochemistry at the Faculty of Science at Masaryk University in Brno, Czech Republic and a group leader at the International Centre for Clinical Research. Research of his group focuses on protein engineering, development of new concepts, microfluidic devices and software tools for protein engineering. He is a co-founder of the first biotechnology spin-off from Masaryk University Enantis Ltd.

**David Bednar** is an associate professor and a leader of a group focused on molecular modelling and bioinformatics within Loschmidt's laboratories at the Faculty of Science at Masaryk University. His research interests are in the development of bioinformatics tools and databases, understanding basic principles of enzymology, and designing mutations to improve protein properties.
