# Supplementary material for: PredictONCO: a web tool supporting decision-making in precision oncology by extending the bioinformatics predictions with advanced computing and machine learning
Source: Brief Bioinform. 2023 Dec 8;25(1):bbad441. doi: 10.1093/bib/bbad441 (PMC10709543; doi:10.1093/bib/bbad441)
Supplement: report_ALK_E1197K_bbad441 [file report_alk_e1197k_bbad441.pdf]

PREDICTONCO v1.0

Job output information

ID: mwxhgu

Title: ALK E1197K

Job submitted: 14 09 2023 18:54

Results accessed: 23 10 2023 13:15

Results link: https://loschmidt.chemi.muni.cz/predictonco/job/mwxhgu

Contents

- Summary for ALK E1197K
- Mutant description
- Catalytic residues
- Conservation
- Inhibitors
- Top scoring inhibitor chart
- Additional information

Summary for **ALK E1197K**

Large Increase · Moderate Increase · Large Decrease · Moderate Decrease · No change · Deleterious

Impact - PredictONCO

Oncogenic

100%

Impact - PredictSNP

Deleterious

87%

Confidence

Stability - FoldX

Deleterious

1.1

-7 0 7

kcal/mol

Stability - Rosetta

Deleterious

6.8

-7 0 7

kcal/mol

Mutation position

Non-essential residue in cytoplasmic domain

Conservation

Moderate evolutionary conservation

Catalytic residues

Changes in pKa

H1124 K1150 E1167 D1249 D1270

Inhibitors

Changes in binding energy

0 70 4172 99 0

Mutant description

HOPE

- There is a difference in charge between the wild-type and mutant amino acid.
- The mutation introduces the opposite charge at this position. This possibly disrupts contacts with other molecules.
- The wild-type and mutant amino acids differ in size.
- The mutant residue is bigger than the wild-type residue.
- The residue is located on the surface of the protein, mutation of this residue can disturb interactions with other molecules or other parts of the protein.

Catalytic residues

show differences show details

E1197K Changes (pKa): H1124 K1150 E1167 D1249 D1270

Conservation

HOPE

- The wild-type residue is very conserved, but a few other residue types have been observed at this position too.
- Neither your mutant residue nor another residue type with similar properties was observed at this position in other homologous sequences. Based on conservation scores this mutation is probably damaging to the protein.

Inhibitors

associated drug

The following table shows the first 50 inhibitors with the best mutant binding energy.

Database	Inhibitor Name	Wild Type [kcal/mol]	Mutant [kcal/mol]	Difference [kcal/mol]
DB11363	Alectinib	-9.8	-9.9	-0.1
DB09063	Ceritinib	-8.2	-8.2	0
DB12267	ZINC148723177	-8.2	-8.1	0.1
DB08865	Xalkori	-8.3	-8.1	0.2
DB12010	Fostamatinib	-7.5	-7.5	0
DB13879	ZINC936069565	-10.4	-10.6	-0.2
ZINC30726863	Cepharanthine	-10.3	-10.5	-0.2
DB09158	Trypan blue	-10.6	-10.5	0.1
DB13345	Ergoloid	-10.2	-10.3	-0.1
DB00320	Dihydroergotamine	-10.2	-10.3	-0.1
DB01126	Avodart	-10.4	-10.3	0.1
DB06469	Lestaurtinib	-10	-10	0
ZINC164528615	ZINC164528615	-10.1	-10	0.1
ZINC14880002	Dihydroergotoxine	-9.9	-9.9	0
DB00762	Irinotecan	-9.6	-9.8	-0.2
DB13941	Piperaquine	-9.8	-9.8	0
DB11630	ZINC3934128	-9.9	-9.8	0.1
ZINC14880001	ZINC14880001	-9.8	-9.8	0
DB11273	ZINC4215648	-9.7	-9.7	0
ZINC118913658	ZINC118913658	-9.7	-9.6	0.1
DB00696	Ergotamine	-9.4	-9.6	-0.2
DB09048	Netupitant	-9.8	-9.6	0.2
DB04868	Nilotinib	-9.6	-9.6	0
DB00197	Troglitazone	-9.7	-9.6	0.1
ZINC11616153	ZINC11616153	-9.8	-9.5	0.3
ZINC119434	Strychnine	-9.6	-9.5	0.1
DB00416	Metocurine	-9.1	-9.5	-0.4
DB11274	Dihydroergocryptine	-9.6	-9.5	0.1
DB13953	Estradiol benzoate	-9.6	-9.5	0.1
DB00210	Differin	-9.6	-9.5	0.1
ZINC40165217	ZINC40165217	-9.4	-9.4	0
ZINC40165218	ZINC40165218	-9.2	-9.4	-0.2
ZINC110344463	2-hydroxyethinylestradiol	-9.3	-9.4	-0.1
ZINC299818022	ZINC299818022	-9.2	-9.4	-0.2
DB11575	Grazoprevir	-8.8	-9.4	-0.6
DB01199	Tubocurarin	-9.5	-9.4	0.1
DB09335	Alatrofloxacin	-9.5	-9.4	0.1
ZINC5167162	6-beta-hydroxyprogesterone	-9.5	-9.4	0.1
DB11275	Epicriptine	-9.4	-9.4	0
ZINC11616152	ZINC11616152	-9.2	-9.3	-0.1
ZINC95617641	Cgp72383	-9.2	-9.3	-0.1
ZINC5764534	6beta-hydroxytestosterone	-9.1	-9.3	-0.2
ZINC77300876	ZINC77300876	-9.6	-9.3	0.3
DB00396	Gesterol	-9.3	-9.3	0
ZINC3917540	ZINC3917540	-9.3	-9.3	0
ZINC33889315	ZINC33889315	-9.3	-9.2	0.1
ZINC13515303	17-alpha-estradiol-3-glucuronide	-9.2	-9.2	0
ZINC95618880	Clofazimine glucuronide	-9.3	-9.2	0.1
ZINC299818021	ZINC299818021	-8.8	-9.2	-0.4
DB00197	Troglitazone	-9.5	-9.2	0.3

Other associated drugs

DB00171, ATP

DB12141, Gilteritinib
DB12130, Lorlatinib

Top scoring inhibitor chart

show only associated drugs

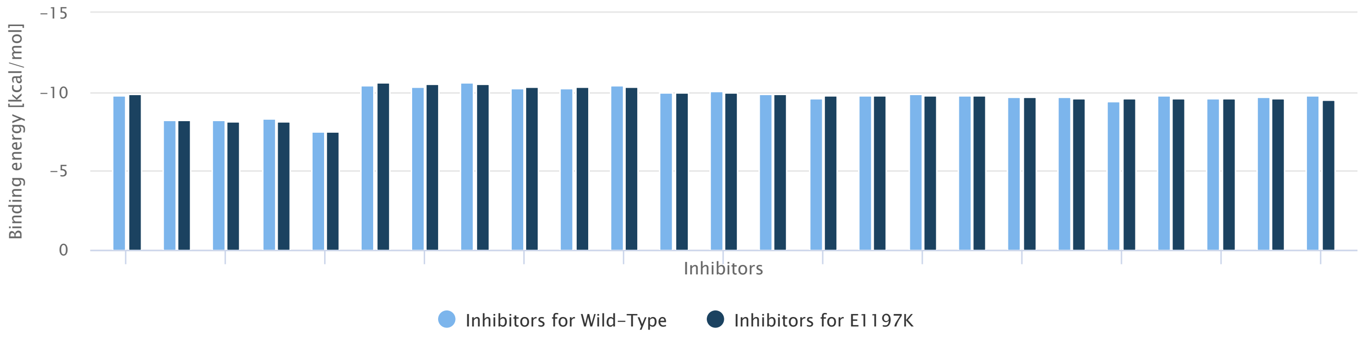

‹ 1 2 3 4 ›

Additional information

UniProtKB

UniProtKB accession for the wild-type protein is Q9UM73. The following information is extracted from the UniProtKB entry.

Function

Neuronal receptor tyrosine kinase that is essentially and transiently expressed in specific regions of the central and peripheral nervous systems and plays an important role in the genesis and differentiation of the nervous system (PubMed:11121404, PubMed:11387242, PubMed:16317043, PubMed:17274988, PubMed:30061385, PubMed:34646012, PubMed:34819673). Also acts as a key thinness protein involved in the resistance to weight gain: in hypothalamic neurons, controls energy expenditure acting as a negative regulator of white adipose tissue lipolysis and sympathetic tone to fine-tune energy homeostasis (By similarity). Following activation by ALKAL2 ligand at the cell surface, transduces an extracellular signal into an intracellular response (PubMed:30061385, PubMed:33411331, PubMed:34646012, PubMed:34819673). In contrast, ALKAL1 is not a potent physiological ligand for ALK (PubMed:34646012). Ligand-binding to the extracellular domain induces tyrosine kinase activation, leading to activation of the mitogen-activated protein kinase (MAPK) pathway (PubMed:34819673). Phosphorylates almost exclusively at the first tyrosine of the Y-x-x-x-Y-Y motif (PubMed:15226403, PubMed:16878150). Induces tyrosine phosphorylation of CBL, FRS2, IRS1 and SHC1, as well as of the MAP kinases MAPK1/ERK2 and MAPK3/ERK1 (PubMed:15226403, PubMed:16878150). ALK activation may also be regulated by pleiotrophin (PTN) and midkine (MDK) (PubMed:11278720, PubMed:11809760, PubMed:12107166, PubMed:12122009). PTN-binding induces MAPK pathway activation, which is important for the anti-apoptotic signaling of PTN and regulation of cell proliferation (PubMed:11278720, PubMed:11809760, PubMed:12107166). MDK-binding induces phosphorylation of the ALK target insulin receptor substrate (IRS1), activates mitogen-activated protein kinases (MAPKs) and PI3-kinase, resulting also in cell proliferation induction (PubMed:12122009). Drives NF-kappa-B activation, probably through IRS1 and the activation of the AKT serine/threonine kinase (PubMed:15226403, PubMed:16878150). Recruitment of IRS1 to activated ALK and the activation of NF-kappa-B are essential for the autocrine growth and survival signaling of MDK (PubMed:15226403, PubMed:16878150)

Subunit interactions

Homodimer; homodimerizes following heparin- and ligand-binding (PubMed:16317043, PubMed:25605972, PubMed:34646012, PubMed:34819673).

Interacts with CBL, IRS1, PIK3R1 and PLCG1 (PubMed:15226403).

Interacts with FRS2 and SHC1 (PubMed:15226403, PubMed:16878150, PubMed:17274988).

Interacts with PTN and MDK (PubMed:11278720, PubMed:12122009).

Publications: PubMed:16878150, PubMed:34819673, PubMed:15226403, PubMed:16317043, PubMed:25605972, PubMed:12122009, PubMed:17274988, PubMed:11278720, PubMed:34646012.

Protein-protein interactions

With	Supporting Experiments	IntAct
itself	10	Q9UM73,Q9UM73
ALKAL1 [Q6UXT8]	7	Q9UM73,Q6UXT8
ALKAL2 [Q6UX46]	5	Q9UM73,Q6UX46
HSP90AB1 [P08238]	2	Q9UM73,P08238
PTPRZ1 [P23471]	2	Q9UM73,P23471
RET [P07949]	2	Q9UM73,P07949

Protein involvement in disease

A chromosomal aberration involving ALK is found in a form of non-Hodgkin lymphoma. Translocation t(2;5)(p23;q35) with NPM1. The resulting chimeric NPM1-ALK protein homodimerize and the kinase becomes constitutively activated. The constitutively active fusion proteins are responsible for

5-10% of non-Hodgkin lymphomas.

A chromosomal aberration involving ALK is associated with inflammatory myofibroblastic tumors (IMTs). Translocation t(2;11)(p23;p15) with CARS; translocation t(2;4)(p23;q21) with SEC31A.

A chromosomal aberration involving ALK is associated with anaplastic large-cell lymphoma (ALCL). Translocation t(2;17)(p23;q25) with ALO17.

The ALK signaling pathway plays an important role in glioblastoma, the most common malignant brain tumor of adults and one of the most lethal cancers. It regulates both glioblastoma migration and growth.

A chromosomal aberration involving ALK is found in one subject with colorectal cancer. Translocation t(2;2)(p23.1;p23.3). A 5 million base pair tandem duplication generates an in-frame WDCP-ALK gene fusion.

A chromosomal aberration involving ALK has been identified in a subset of patients with non-small-cell lung carcinoma. This aberration leads to the production of a fusion protein between the N-terminus of EML4 et the C-terminus of ALK. It is unclear whether the fusion protein is caused by a simple inversion within 2p (inv(2)(p21p23)) or whether the chromosome translocation involving 2p is more complex. When tested in a heterologous system, the fusion protein EML4-ALK possesses transforming activity that is dependent on ALK catalytic activity, possibly due to spontaneous dimerization mediated by the EML4 moiety, leading to ALK kinase activation.

Neuroblastoma 3 (NBLST3)

Publications: PubMed:21242967, PubMed:18724359, PubMed:18923523, PubMed:18923525, PubMed:22932897.

Disease susceptibility is associated with variants affecting the gene represented in this entry.

Disease description: A common neoplasm of early childhood arising from embryonic cells that form the primitive neural crest and give rise to the adrenal medulla and the sympathetic nervous system.

GO - Molecular function

- GO:0005524 - ATP binding
- GO:0008201 - heparin binding
- GO:0042802 - identical protein binding
- GO:0004704 - NF-kappaB-inducing kinase activity
- GO:0004713 - protein tyrosine kinase activity
- GO:0030298 - receptor signaling protein tyrosine kinase activator activity
- GO:0004714 - transmembrane receptor protein tyrosine kinase activity

GO - Biological process

- GO:0030534 - adult behavior
- GO:0097009 - energy homeostasis
- GO:0021766 - hippocampus development
- GO:0050995 - negative regulation of lipid catabolic process
- GO:0048666 - neuron development
- GO:0038083 - peptidyl-tyrosine autophosphorylation
- GO:0016310 - phosphorylation
- GO:1900006 - positive regulation of dendrite development
- GO:0033674 - positive regulation of kinase activity
- GO:0051092 - positive regulation of NF-kappaB transcription factor activity
- GO:0046777 - protein autophosphorylation
- GO:0042981 - regulation of apoptotic process
- GO:0042127 - regulation of cell population proliferation
- GO:0060159 - regulation of dopamine receptor signaling pathway
- GO:0045664 - regulation of neuron differentiation
- GO:0090648 - response to environmental enrichment
- GO:0007165 - signal transduction
- GO:0036269 - swimming behavior
- GO:0007169 - transmembrane receptor protein tyrosine kinase signaling pathway
